# PREGABALIN AS A PREOPERATIVE ADJUVANT IN PATIENTS WITH CARPAL TUNNEL SYNDROME

**DOI:** 10.1590/1413-785220243204e278895

**Published:** 2024-10-07

**Authors:** Fábio Hideki Nishi Eto, Thiago Broggin Dutra Rodrigues, Victor Elzio Gasperoni Matias, Yussef Ali Abdouni

**Affiliations:** 1. Irmandade Santa Casa de Misericordia de Sao Paulo, Departamento de Ortopedia e Traumatologia “Pavilhão Fernandinho Simonsen”,Sao Paulo, SP, Brazil

**Keywords:** Carpal Tunnel Syndrome, Surgical Procedures, Operative Pregabalin, Pain, Síndrome Do Túnel Do Carpo, Procedimento Cirúrgico, Pregabalina, Dor

## Abstract

**Objective:**

To evaluate the pregabalin adjuvant effect in patients with carpal tunnel syndrome (CTS) surgically treated, analyzing postoperative pain and the incidence of complex regional pain syndrome (CRPS).

**Methods:**

Outpatient surgical candidates with CTS were selected and followed for 12 months, divided into three groups. The Control Group received a placebo, the Pregabalin 75mg Group received a daily dose, and the Pregabalin 150mg Group received a daily dose of the medication. Patient progress was evaluated using the visual analog scale (VAS) for pain and the DN4 neuropathic pain score before surgery, one month and three months after.

**Results:**

The administration of pregabalin to surgical patients with CTS did not demonstrate significant differences in immediate postoperative pain relief. Additionally, there were no statistically significant variations in the incidence of complications, such as CRPS, among the groups.

**Conclusion:**

This study did not show a significant impact of pregabalin on postoperative pain relief or the reduction of CRPS incidence in patients undergoing surgery for CTS. These results suggest that pregabalin might not be an effective adjuvant in these surgical situations. ***Level of evidence II (Oxford), Prospective Comparative Study.***

## RESUMO

**Objetivo:**

*Avaliar o efeito adjuvante da pregabalina em pacientes com síndrome do túnel do carpo (STC) tratados cirurgicamente, analisando a dor pós-operatória e a incidência da síndrome da dor complexa regional (SDCR).*

**Métodos:**

*Foram selecionados pacientes com acompanhamento ambulatorial e indicação de tratamento cirúrgico para STC, sendo acompanhados ao longo de 12 meses e divididos em três grupos. O Grupo Controle recebeu placebo, o Grupo Pregabalina 75 mg tomou uma dose diária da medicação citada e o Grupo Pregabalina 150 mg também recebeu uma dose diária da medicação, em maior quantidade. A evolução dos pacientes foi avaliada mediante aplicação da escala visual analógica de dor (EVA) e escore de dor neuropática DN4 antes da cirurgia, um mês e três meses após essa.*

**Resultados:**

*A administração de pregabalina em pacientes cirúrgicos com STC não demonstrou diferenças significativas no alívio da dor pós-operatória imediata. Além disso, não houve variações estatisticamente significativas na incidência de complicações, como a SDCR, entre os grupos.*

**Conclusão:**

*Este estudo não evidenciou um impacto significativo da pregabalina no alívio da dor pós-operatória ou na redução da incidência da SDCR em pacientes submetidos a cirurgia para STC. Estes resultados sugerem que a pregabalina pode não ser um adjuvante eficaz nessas situações cirúrgicas. **Nível II de Evidência (Oxford), Estudo prospectivo comparativo.***

## INTRODUCTION

 Carpal tunnel syndrome (CTS) is the most common compressive upper limb neuropathy, affecting approximately 4% of the general population, and it is more prevalent in females and between 45 and 60 years of age. ^1^ The clinical status is characterized by pain and paresthesia in the territory of the median nerve, with an insidious onset and, in the most severe cases, loss of strength and atrophy of the thenar muscles is observed. ^2^ The carpal tunnel is an osteofibrous, inelastic canal, whose roof is the transverse carpal ligament, and nine tendons and the median nerve pass through it. 

 Surgical treatment for CTS consists of releasing the transverse ligament, leading to the nerve decompression. ^3^ Despite being a widely performed procedure in hand surgery, with high success rates, surgery for treating CTS may present unsatisfactory results for the patient. Many complications cannot be prevented, such as the development of chronic postoperative pain. ^4^

 Complex regional pain syndrome (CRPS) is divided into CRPS type 1 (formerly known as reflex sympathetic dystrophy) and CRPS type 2 (formerly known as causalgia) and is a chronic pain condition with neuropathic characteristics, generally of disproportional intensity to the nociceptive stimulus. Presence of vasomotor changes may or may not be associated. ^5^ There is a predominance in females and there is no evidence that risk factors predispose to the CRPS development, although immobilization for a prolonged period of time may act as a predisposing factor. Its incidence after carpal tunnel decompression surgery, regardless of the technique, is around 8% ^6^ and, in some series of cases, it corresponds to half of the complications after this type of procedure. ^7^

 Recently, with the understanding of the central sensitization processes that lead to chronic pain, drugs from the gabapentinoid class, especially pregabalin, started being studied in order to prevent CRPS. Most of studies found in the literature analyze the reduction in pain scores and opioid consumption in knee surgeries when pregabalin was used preemptively. ^8^^,^^9^ However, there is still no consensus on the dose or the length of time these medications should be used. The objective of this study is to evaluate the effectiveness of pregabalin as a preoperative adjuvant in carpal tunnel decompression surgeries. 

## MATERIALS AND METHODS

 In this study, patients with a diagnosis of CTS, treated at the outpatient clinic of the *Grupo da Mão e Microcirurgia* at *Santa Casa de Misericórdia de São Paulo* , from June 2022 to June 2023, were evaluated and followed prospectively. The research was conducted upon approval by the Ethics and Research Committee of the aforementioned institution, following resolution 196/96 (CAAE: 69653223.9.0000.5479), and all patients signed the Informed Consent Form (ICF). 

These patients were randomly subdivided into three groups:

- Patients who received placebo during the three weeks before surgery.
- Patients who received 75 mg/day of pregabalin during the three weeks before surgery.
- Patients who received 150 mg/day of pregabalin during the three weeks before surgery.

Patients of both sexes, aged between 40 and 70 years, who received a confirmed diagnosis of CTS using any of the following methods were included in this study: ultrasound, electroneuromyography, or clinical examination. In this study, surgical median nerve decompression was performed by the same surgeon, using the mini-incision surgical technique. Patients who had previous surgeries on the same hand, other associated neuropathies, previous use of gabapentinoids, and a history of CRPS were excluded from the study.

The data were collected and analyzed by the same researcher, through the use of the Pain Visual Analogue Scale (VAS) and evaluation of the DN4 questionnaire for neuropathic pain. All patients adopted the same follow-up protocol. Patients started being given the medication or placebo three weeks before the procedure, and assessments were made in the immediate postoperative period, as well as one month and three months after surgery. All patients used an orthosis for immobilization until the surgical wound stitches were removed, which occurred two weeks after surgery. In addition, they received simple analgesia with nonsteroidal anti-inflammatory drugs (NSAIDs) and tramadol for pain relief when necessary in the first two weeks. They were also monitored by the occupational therapy team until the last assessment.

 Patients were assessed before the procedure, one month and three months after surgery. The assessed quantitative characteristics were described according to groups using summary measures (means, standard deviations, medians, and quartiles) and compared between groups using analysis of variance (ANOVA) or Kruskal-Wallis test, and the qualitative characteristics were described according to groups using absolute and relative frequencies, with verification of the association by the likelihood ratio test. ^10^

 Pain scores were described according to groups throughout the moments evaluated using summary measures and compared between groups and moments using generalized estimating equations (GEE) with normal marginal distribution and identity link function, assuming a first-order autocorrelation coefficient (AR(1)) between the assessment moments. ^11^ The analyses were followed by Bonferroni multiple comparisons%5E@12 ^ to verify between which groups and moments the differences occurred. 

The analyses were performed using IBM-SPSS for Windows version 22.0 and tabulated using Microsoft-Excel 2013; the tests were carried out with a 5% significance level.

## RESULTS

Of the 45 patients diagnosed with CTS and indicated for surgical treatment, 18 patients composed group 1 (placebo during the three weeks

before surgery), 15 patients formed group 2 (pregabalin 75 mg/day during the three weeks before surgery), and 13 patients formed group 3 (pregabalin 150 mg/day during the three weeks before surgery). Four patients were excluded from the study due to previous use of gabapentin, loss of postoperative follow-up, and non-adherence to the medication proposed before surgery.

 Both VAS and DN4 showed a statistically similar average behavior of groups throughout the assessment moments (p Interaction > 0.05); VAS showed a difference between groups regardless of the assessment moment (p Group = 0.007), and VAS and DN4 showed differences on average throughout the assessment’s moments regardless of group (p Moment < 0.05) ( Table 1 ). 

 The VAS score was higher in the Pregabalin 75 mg group than in the placebo group regardless of the assessment moment (p = 0.006), and both VAS and DN4 decreased from the preoperative period to the other moments, regardless of the group (p < 0.001). With this kind of statistical result evaluation, it became clear that, regardless of the group, the patients presented similar results with a reduction in VAS and an improvement in DN4 in postoperative assessments. Furthermore, the occurrence of CRPS was not evident until three months after surgery in any of the three groups ( Table 2 ). 

 The other demographic data can be seen in Table 3 . 

**Table 1.** Description of pain scores according to groups throughout the assessment moments and results of comparisons

Variable/Moment	Group			P Grup	P moment	P Interaction
	Placebo	Pregabalin 75 mg	Pregabalin 150 mg			
**VAS**				**0.007**	**<0.001**	0.166
**Pre-op**						
Mean ± SD	7.9 ± 2	8.8 ± 1.8	8.4 ± 1.5			
Median (p25; p75)	8 (6.3; 10)	10 (8; 10)	9 (7; 10)			
**1 month**						
Mean ± SD	1.7 ± 2.4	2.9 ± 3.8	2.1 ± 1.8			
Median (p25; p75)	0 (0; 4,8)	0 (0; 6.5)	2 (0; 4)			
**3 months**						
Mean ± SD	0.4 ± 0.9	3.5 ± 2.8	0.8 ± 1.5			
Median (p25; p75)	0 (0; 0)	3 (1; 6.5)	0 (0; 2)			
**DN4**				0.375	**<0.001**	0.456
**Pre-op**						
Mean ± SD	5.3 ± 2.1	6.2 ± 1.5	6 ± 2.4			
Median (p25; p75)	4.5 (4; 7)	7 (5; 7.5)	7 (5; 8)			
**1 month**						
Mean ± SD	1.3 ± 1.3	1.5 ± 2	1.8 ± 2			
Median (p25; p75)	1 (0; 2)	1 (0; 2.5)	1 (0; 4)			
**3 months**						
Mean ± SD	0.6 ± 1.1	1.6 ± 2	0.9 ± 1.3			
Median (p25; p75)	0 (0; 1)	1 (0; 3)	0 (0; 2)			

EEG with normal distribution and identity connection function, assuming correlation matrix AR(1) between the moments

**Table 2.** Score comparison

Variable	Comparison	Average difference	Standard error	p	CI (95%)	
					Inferior	Superior
VAS	Placebo – Pregabalin 75 mg	-1.74	0.56	0.006	-3.09	-0.39
	Placebo – Pregabalin 150 mg	-0.45	0.59	>0.999	-1.86	0.97
	Pregabalin 75 mg – Pregabalin 150mg	1.29	0.62	0.110	-0.19	2.78
	Pre-op – 1 month	6.13	0.42	<0.001	5.12	7.14
	Pre-op – 3 months	6.76	0.48	<0.001	5.62	7.90
	1 month – 3 months	0.63	0.42	0.406	-0.38	1.64
DN4	Pre-op – 1 month	4.27	0.26	<0.001	3.66	4.89
	Pre-op – 3 months	4.78	0.32	<0.001	4.00	5.55
	1 month – 3 months	0.51	0.26	0.146	-0.11	1.12

Multiple Bonferroni Comparisons

**Table 3.** Epidemiological data

Variable	Group			Total (N=40)	p
	Placebo (N=16)	Pregabalin 75 mg (N=13)	Pregabalin 150 mg (N=11)		
**Age (years)**					0.162**
Mean ± SD	61 ± 14.8	51.5 ± 8.2	56.2 ± 14.7	56.6 ± 13.3	
Median (p25; p75)	60 (47.5; 75.8)	54 (47; 57.5)	52 (41.5; 71.5)	55 (41; 69)	
**Gender**					0.088
Female	15 (93.8)	12 (92.3)	7 (63.6)	34 (85)	
Male	1 (6.3)	1 (7.7)	4 (36.4)	6 (15)	
**Side**					0.031
Right	7 (43.8)	5 (38.5)	1 (9.1)	13 (32.5)	
Left	4 (25)	5 (38.5)	1 (9.1)	10 (25)	
Bilateral	5 (31.3)	3 (23.1)	9 (81.8)	17 (42.5)	
**Time of symptoms (years)**					0.907£
Mean ± SD	2.7 ± 2.5	2.6 ± 2.4	2.5 ± 2.5	2.6 ± 2.4	
Median (p25; p75)	2 (1; 3)	2 (1.5; 3)	2 (0.7; 3)	2 (1; 3)	

Likelihood ratio test

** Unpaired Student’s T test

£ Kruskal-Wallis test

## DISCUSSION

 Pregabalin acts to modulate calcium channels present in neurons, showing proven effects as an antiepileptic and anxiolytic agent, in addition to acting as an analgesic in situations of neuropathic pain. ^13^ These results supported the inclusion of such substance in this study, with the aim of reproducing and analyzing its effects on the postoperative period of patients with an already established diagnosis of CTS. 

All assessed patients were already candidates for surgical treatment for CTS due to failure of clinical treatment or due to muscular hypotrophy in the thenar region, and, although gabapentinoids are approved for the treatment of chronic neuropathic pain, this medication has not yet been proven as being effective in managing CTS postoperative pain.

 In a study carried out by Sadatsun, ^14^ it was found that the use of gabapentin, an anticonvulsant with similar action to pregabalin, in a single dose of 600 mg, one hour before anesthetic induction, did not present significant results in patients with CTS. A result similar to that found in this study. Even using the medication for one month throughout the preoperative period, few patients reported improvement in symptoms before surgery with the use of the medication, not avoiding the procedure. 

 Other studies, however, demonstrate that the use of gabapentinoids allowed the reduction of the use of other medications, such as opioids, in the management of major surgical procedures, ^8^ but this variable was not evaluated in this study, since the assessed patients maintained regular use of analgesics in postoperative follow-up. 

Another aspect that must be taken into consideration in this study is that all patients already had surgical indications before the medication was administrated and, as there was no statistical difference between the control group and the medication groups, we raised the hypothesis that patients who have already more severe CTS, or are refractory to conservative treatment, did not obtain any advantages when operated in association with medication, with the improvement being attributed to the surgical procedure itself. Another aspect that requires consideration in this study is that all patients already had surgical indications before the medication administration. As no statistically significant difference was found between the control group and the groups that received the medication under analysis, this situation raises the hypothesis that patients who already have a more severe CTS condition and who do not respond well to non-surgical treatment do not seem to benefit from concomitant treatment between pregabalin and the surgical procedure, with the improvement in the condition being mainly attributed to the surgery.

Regarding CRPS, although patients did not manifest this condition during the study period, the literature reports an incidence of approximately 8% of this condition in patients with CTS. Therefore, a study with a larger sample of patients could reveal other outcomes.

## CONCLUSION

During the period assessed, no significant difference was found with the use of pregabalin in relation to the pain experienced by the patient upon application of the VAS and DN4, nor in terms of the occurrence of CRPS.
